# *Helicobacter pylori* infection is not an independent risk factor of non-alcoholic fatty liver disease in China

**DOI:** 10.1186/s12876-022-02148-6

**Published:** 2022-02-24

**Authors:** Weijun Wang, Mengke Fan, Rui Gong, Yurui Zhang, Junchao Zeng, Sanping Xu, Rong Lin

**Affiliations:** 1 Department of Gastroenterology, Union Hospital, Tongji Medical College, Huazhong University of Science and Technology, Wuhan, 430022 China; 2 Health Management Center, Union Hospital, Tongji Medical College, Huazhong University of Science and Technology, Wuhan, 430022 China

**Keywords:** *H. pylori*, NAFLD, Risk factor, Infection, Association

## Abstract

**Background:**

The role of *Helicobacter pylori* (*H. pylori*) infection in the development of non-alcoholic fatty liver disease (NAFLD) remains controversial. The exact relationship requires further investigation. This study aimed to determine the association between them in China.

**Methods:**

A retrospective study was conducted on 71,633 participants who underwent physical examinations. ^13^C urea breath test (^13^C-UBT) was conducted to detect *H. pylori* infection, and ultrasonography was used to detect NAFLD.

**Results:**

Body mass index (BMI), blood pressure (BP), and triglyceride (TG) levels were higher in participants with *H. pylori* infection than in those without *H. pylori* infection. While the levels of high-density lipoprotein cholesterol (HDL-C) for participants with *H. pylori* infection was lower than without *H. pylori* infection (*P* < 0.001). After adjusting for confounding factors (age, sex, BMI, BP, Scr, BUN, LDL-C, HDL-C, triglycerides, FBG and HbA1c), multivariate logistic regression analysis indicated that there was no independent relationship between them (*P* = 0.574). Subgroup analysis (stratified by sex, age, BMI, hypertension, diabetes and dyslipidemia) showed that *H. pylori* infection was not included as an independent risk factor for NAFLD. Moreover, the different grades of NAFLD were not related to *H. pylori* infection.

**Conclusions:**

These results indicate that *H. pylori* infection is not an independent risk factor for NAFLD in China.

**Supplementary Information:**

The online version contains supplementary material available at 10.1186/s12876-022-02148-6.

## Background

Non-alcoholic fatty liver disease (NAFLD), manifested by hepatic steatosis without alcohol consumption or other causes of liver disease, is an important form of chronic liver disease [1]. It is increasingly recognized that NAFLD can cause both liver disease and extrahepatic manifestations, including obesity, dyslipidemia, diabetes, insulin resistance, chronic kidney disease and extrahepatic malignancies [2–5]. Currently, the prevalence of NAFLD is rapidly increasing, contributing to enormous clinical and financial burden [1]. Therefore, it is of great importance to identify risk factors that may have therapeutic implications for the prevention and treatment of NAFLD, as well as the associated burden.

Various mechanisms related to the intestinal microbiome and NAFLD have been proposed, such as its impact on the innate immune system, intestinal endothelial barrier function, intestinal production of metabolites, and fermentation of indigestible carbohydrates [6, 7]. Animal experiments also suggest that intestinal dysbiosis or *Helicobacter pylori* (*H. pylori*) infection may lead to the occurrence and development of NAFLD [8]. *H. pylori* is a gram-negative bacterium that colonizes the gastric epithelium [9]. It is a key element of the human microbiome. Although current evidence is not definitive, chronic *H. pylori* infection has been demonstrated to be related to inflammatory bowel diseases (IBD), gastrointestinal cancers, and extra-digestive tract diseases, including cardiovascular, pulmonary, hematological, ophthalmic, skin, neurological, and metabolic diseases [10, 11]. A previous study reported that *H. pylori* infection may lead to gastrointestinal flora dysbiosis, increase the levels of inflammatory cytokines, promote insulin resistance, and accelerate fatty deposits in the liver tissue, which contribute to NAFLD [12].

There have been increasing numbers of studies investigating the association of *H. pylori* and NAFLD [13, 14]. Several researches have shown that *H. pylori* infection is related to the development of NAFLD, regardless of inflammatory and metabolic risk factors [13]. On the contrary, other retrospective studies indicated that in apparently healthy subjects, *H. pylori* infection is not an independent risk factor for patients suffering from NAFLD [14]. To clarify this inconsistency, it is necessary to further investigate the association between *H. pylori* and NAFLD.

A broad population-based study may help clarify the relationship between these factors. This cross-sectional study of 71,633 participants recruited from the Wuhan Union Hospital aimed to determine the association between *H. pylori* infection and different grades of NAFLD (total/mild/moderate/severe). Meanwhile, the role of *H. pylori* in NAFLD was further studied in subgroups, characterized by sex, age, body mass index (BMI), hypertension, diabetes, and dyslipidemia.

## Methods

### Study population

The cross-sectional study included healthy adults who underwent comprehensive medical checkups at the Wuhan Union Hospital, from January 2015 to December 2019. Participants with both abdominal ultrasound (US) for the detection of liver steatosis and ^13^C urea breath test (^13^C-UBT) for the detection of *H. pylori* infection were included (n = 122,764). Participants with a self-reported history of other chronic liver diseases (viral hepatitis, autoimmune hepatitis, etc.), daily alcohol consumption (male: > 30 g; female: > 20 g); positive markers for hepatitis A, B, or C virus; self-reported history of malignancy; and missing data on basic information were excluded from the study. In total, 71,633 healthy participants were included in the analysis. The chart of this study is presented in Additional file 1: Fig. S1. Table 1 shows the baseline characteristics of the participants. This study was approved by the Wuhan Union Hospital Ethics Committee and the Ethics Committee of Tongji Medical College, Huazhong University of Science and Technology (IORG No: IORG0003571). Informed consent was waived by the ethics committees.

**Table 1:** Characteristics of study participants at baseline

Variables	Full sample (n = 71,633)			Matched sample (n = 29,974) ^a^		
	*H. pylori−* (n = 46,888)	*H. pylori* + (n = 24,745)	*P* value	*H. pylori*− (n = 14,987)	*H. pylori* + (n = 14,987)	*P* value
Age	44.8 ± 13.3	46.5 ± 12.5	< 0.001	47.3 ± 12.9	46.6 ± 12.2	
Male (%)	57.6	58.6	0.010	58.3	57.5	0.134
BMI (kg/m^2^)	23.8 ± 3.4	24.1 ± 3.4	< 0.001	24.3 ± 3.3	24.2 ± 3.4	0.043
Year of screening exam (%)			< 0.001			
2015	20.8	21.7		21.6	12.6	
2016	24.4	24.2		48.1	27.1	
2017	19.3	18.7		25.1	23.0	
2018	19.8	21.3		4.8	24.5	
2019	18.8	17.3		0.4	12.7	
Systolic BP (mmHg)	124.3 ± 19.5	125.9 ± 20.0	< 0.001	127.1 ± 19.8	126.4 ± 20.0	
Diastolic BP (mmHg)	77.8 ± 11.4	78.9 ± 11.8	< 0.001	79.7 ± 11.5	79.1 ± 11.8	
Hypertension (%)	13.7	15.1	< 0.001	13.5	13.9	0.129
FBG (mM)	5.05 ± 1.27	5.18 ± 1.47	< 0.001	5.11 ± 1.29	5.12 ± 1.29	
HbA1C (%)	5.48 ± 0.75	5.55 ± 0.83	< 0.001	5.50 ± 0.77	5.50 ± 0.77	
Diabetes (%)	4.74	6.14	< 0.001	5.49	5.60	0.565
Total cholesterol (mM)	4.80 ± 0.92	4.86 ± 0.92	< 0.001	4.89 ± 0.91	4.86 ± 0.92	
LDL-C (mM)	2.76 ± 0.77	2.82 ± 0.78	< 0.001	2.22 ± 0.77	2.82 ± 0.77	
HDL-C (mM)	1.43 ± 0.34	1.41 ± 0.34	< 0.001	1.46 ± 0.35	1.41 ± 0.35	
Triglycerides (mM)	1.58 ± 1.39	1.65 ± 1.50	< 0.001	1.65 ± 1.41	1.64 ± 1.44	
Dyslipidemia (%)	24.9	28.0	< 0.001	26.8	26.9	0.887
AST (IU/L)	23.3 (18–25)	23.4 (18–25)	0.011	22.7 (17–25)	23.3 (18–26)	
ALT (IU/L)	26.5 (15–31)	27.0 (15–31)	< 0.001	27.4 (15–32)	27.0 (15–31)	
GGT (IU/L)	30.2 (14–33)	31.0 (15–35)	< 0.001	32.6 (15–36)	30.5 (14–35)	
Scr (μM)	70.5 ± 17.2	71.1 ± 20.1	0.001	69.6 ± 18.6	70.2 ± 17.6	
BUN (mM)	4.88 ± 1.30	4.95 ± 1.37	< 0.001	4.99 ± 1.32	4.94 ± 1.32	
NAFLD (%)	31.3	34.8	< 0.001	34.8	34.6	0.716

Continuous variables were shown as mean ± SD or median (interquartile range), and categorical variables were expressed as counts (percentage)

^a^Matched sample were selected with propensity score matching analysis (PSM) (Covariate: Age, sex, BMI, Systolic BP, Diastolic BP, FBG, HbA1C, Total cholesterol, LDL-C, HDL-C, Triglycerides, Scr, BUN; Matching tolerance: 0.001)

*H. pylori*, *Helicobacter pylori*; BMI, body mass index; BP, blood pressure; FBG, fasting blood glucose; HbA1C, glycated hemoglobin; LDL-C, low-density lipoprotein cholesterol; HDL-C, high-density lipoprotein cholesterol; AST, aspartate aminotransferase; ALT, alanine aminotransferase; GGT, gamma-glutamyl transferase; Scr, serum creatinine; BUN, blood urea nitrogen; NAFLD, nonalcoholic fatty liver disease

### Data collection

Clinical examination data comprised demographic features, anthropometry, laboratory examination, image examination, and a self-administered health questionnaire. The weight, height, and blood pressure were measured by three experienced physicians. Blood samples were collected from the elbow vein after an overnight fast by three experienced nurses. Fasting total cholesterol (TC), low-density lipoprotein cholesterol (LDL), high-density lipoprotein cholesterol (HDL), triglyceride, aspartate aminotransferase (AST), alanine aminotransferase (ALT), γ-glutamyl transpeptidase (GGT), serum creatinine, and urea nitrogen were measured using an automatic biochemical analyzer. Plasma glucose was analyzed using the glucose oxidase method. Glycosylated hemoglobin (HbA1c) levels were measured using high-performance liquid chromatography [15].

### Diagnosis of *Helicobacter pylori* infection

*H. pylori* infection status was diagnosed using ^13^C-UBT. After fasting overnight, each participant ingested an oral pill labeled with the radiocarbon-13 isotope. Respiratory samples were stored at room temperature and evaluated using ^13^CO_2_. *H. pylori* infection was determined by comparing the ^13^CO_2_ content of the baseline and 30-min samples, and a ratio > 4.0 was considered positive. This was based on the Fourth Chinese National Consensus Report on the management of *H. pylori* infection [16].

### Diagnosis of NAFLD

NAFLD was diagnosed by hepatic ultrasonography in the absence of excessive alcohol intake and viral or autoimmune hepatitis. Ultrasonographic examinations were carried out by five experienced specially trained doctors. NAFLD was diagnosed according to the standard USS criteria. Liver steatosis was defined as having at least two of the following three abnormal manifestations: echogenicity enhancement of the liver compared with spleen or kidney; ultrasound beam attenuation; poor visualization of intrahepatic architectural details [17, 18]. In addition, NAFLD was also divided into three different grades (mild, moderate, and severe) according to the Chinese Ultrasonic Grading Criteria of NAFLD published in 2003.

### Diagnosis of hypertension, diabetes, and dyslipidemia

Hypertension was defined as SBP ≥ 140 mmHg or DBP ≥ 90 mmHg [19]. Patients with fasting blood glucose (FBG) ≥ 7.0 mmol/L were defined as having diabetes [20]. Dyslipidemia was confirmed by increases in total cholesterol (≥ 240 mg/dL [6.20 mmol/L]), LDL-C (> 160 mg/dL [4.13 mmol/L]), and triglyceride levels (> 200 mg/dL [2.25 mmol/L]), or a decrease in HDL-C (< 40 mg/dL [1.03 mmol/L]) [21].

### Statistical analysis

Statistical analyses were performed using SPSS, version 26.0 (Chicago, Illinois, USA). Continuous variables were shown as mean ± SD, and categorical variables were expressed as counts (percentage). The Mann–Whitney *U* test and x^2^ test were used to compare the differences between the two groups. Propensity score matching (PSM) analysis was also conducted. Logistic regression analysis was used to assess the association between the factors of interest. And multicollinearity test was performed before multivariate regression analysis. *P* values < 0.05 (two-tailed) were considered statistically significant.

## Results

### Clinical and demographic characteristics

Among the 71,633 involved study participants, 30,086 (42.0%) were female and 41,547 (58.0%) were male. Among all subjects, the prevalence of *H. pylori* infection in this study was 34.5% (n = 24,745). Table 1 summarizes the clinical and biochemical characteristics of the participants based on their *H. pylori* infection status. Males with a high BMI (*H. pylori*− vs. *H. pylori*+ : 23.8 ± 3.4 vs. 24.1 ± 3.4) and high blood pressure (systolic BP: 124.3 ± 19.5 vs. 125.9 ± 20.0; diastolic BP: 77.8 ± 11.4 vs. 78.9 ± 11.8) were more likely to be *H. pylori* positive. Compared with the *H. pylori*− group, the *H. pylori*+ group had a more adverse lipid profile, including higher levels of TC (*H. pylori*− vs. *H. pylori*+: 4.80 ± 0.92 vs. 4.86 ± 0.92), TG (*H. pylori*− vs. *H. pylori*+: 1.58 ± 1.39 vs. 1.65 ± 1.50), and LDL-C (*H. pylori*− vs. *H. pylori*+: 2.76 ± 0.77 vs. 2.82 ± 0.78), and a lower level of HDL-C (*H. pylori*− vs. *H. pylori*+: 1.43 ± 0.34 vs. 1.41 ± 0.34). Moreover, the serum ALT level (*H. pylori*− vs. *H. pylori*+: 26.5 (15–31) vs. 27.0 (15–31)), and GGT (*H. pylori*− vs. *H. pylori*+: 30.2 (14–33) vs. 31.0 (15–35)) in the *H. pylori*+ group were higher than those in the *H. pylori*− group. Moreover, the FBG level (*H. pylori*− vs *H. pylori*+: 5.05 ± 1.27 vs. 5.18 ± 1.47), and HbA1c (*H. pylori*− vs. *H. pylori*+: 5.48 ± 0.75 vs. 5.55 ± 0.83) were higher in the *H. pylori*+ group (Table 1). In the matched samples (n = 29,974), there was no difference in the incidence of hypertension (*P* = 0.129), diabetes (*P* = 0.565), or dyslipidemia (*P* = 0.887) between the two groups (*H. pylori*− and *H. pylori* +). Further analysis showed that there was no significant difference in the incidence of NAFLD between the *H. pylori−* and *H. pylori*+ groups (*P* = 0.716) (Table 1).

### The prevalence of NAFLD and *H. pylori*

**Fig. 1:**
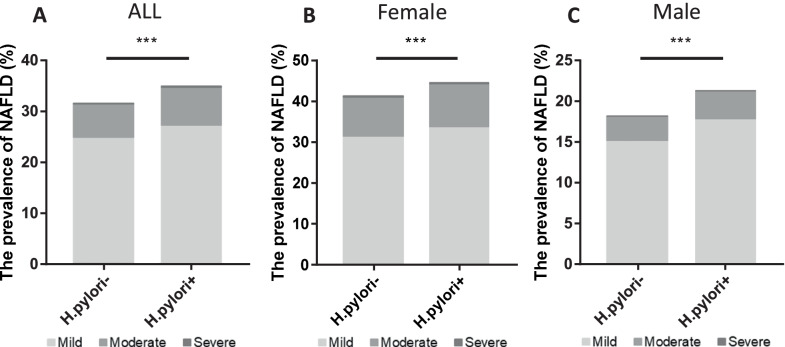
The Prevalence of NAFLD with different H. pylori infectious status in all subjects (**A**), female (**B**), male (**C**). *P < 0.001

The prevalence of NAFLD and the *H. pylori* infection status are shown in Fig. 1. The prevalence of NAFLD in the *H. pylori*+ group (total: 34.8%; mild: 27.1%, moderate: 7.5%, severe: 0.2%) was significantly higher than that in the *H. pylori−* group (total: 31.3%; mild: 24.6%, moderate: 6.5%, severe: 0.2%) (*P* < 0.001). Further stratified analysis by sex revealed that the prevalence of NAFLD with *H. pylori*+ in males was 44.4% (mild: 33.7%, moderate: 10.4%, severe:0.3%), which was higher than that in the *H. pylori*− group (total: 41.1%; mild: 31.7%, moderate: 9.2%, severe: 0.3%) (*P* < 0.001). Moreover, the prevalence of NAFLD in female with *H. pylori*+ (total: 21.1%, mild: 17.7%, moderate: 3.3%, severe: 0.07%) was significantly higher than *H. pylori*− group (total: 17.9%, mild: 15.0%, moderate: 2.8%, severe: 0.03%) (*P* < 0.001).

### Associations between NAFLD and *H. pylori* infection status

**Fig. 2:**
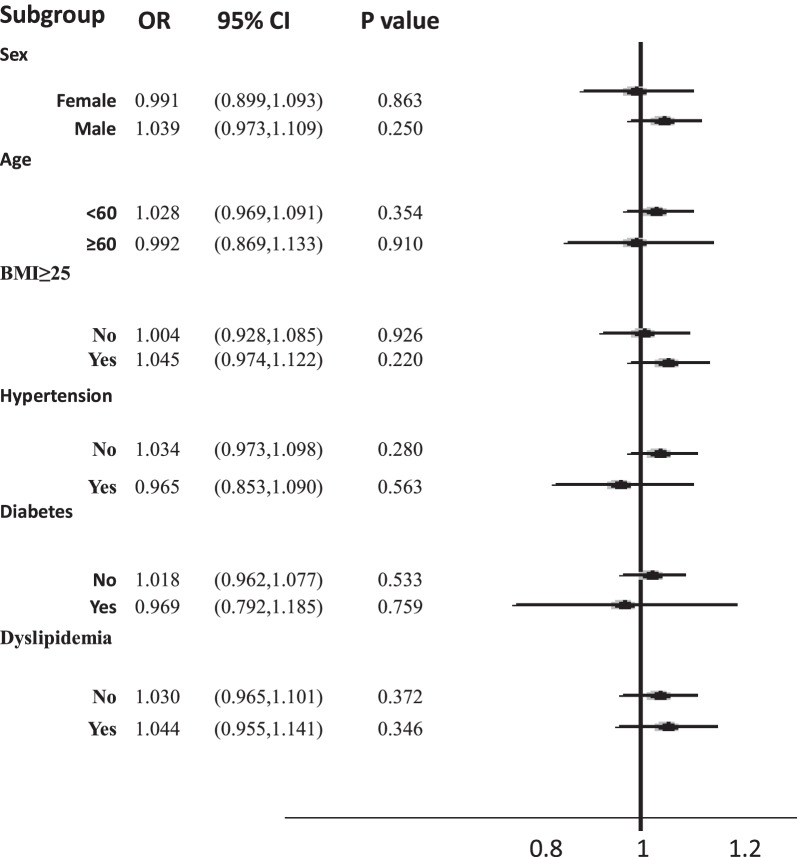
Association between H. pylori and NAFLD in clinically relevant subgroups. Logistic regression analyses were conducted. Confounding factors were adjusted. OR, odds ratios; CI, confidence intervals; BMI, body mass index

The relationship between *H. pylori* infection and NAFLD prevalence was analyzed using logistic regression analysis. In the full samples (n = 71,633), univariate analysis showed that *H. pylori* infection increased the risk of NAFLD (OR = 1.172, 95% CI = 1.135–1.211, *P* < 0.001) (Table 2). Subsequently, we performed a multicollinearity analysis with the significant variables (variance inflation factor test, VIF) and found that there was multicollinearity between total cholesterol (VIF = 13.778) and LDL-C (VIF = 10.511). In contrast, there was no multicollinearity among the other variables (VIF < 10; triglyceride, VIF = 3.968; FBG, VIF = 3.377; HbA1c, VIF = 3.486). We then excluded total cholesterol from the multivariate regression model. The multivariate analysis showed no significant association between *H. pylori* infection and NAFLD (OR = 1.022, 95% CI = 0.967–1.079, *P* = 0.446) (Table 2). In the matched samples (n = 29,974), the univariate analysis (OR = 1.01, *P* = 0.716) and the multivariate analysis (OR = 1.00, *P* = 0.898) showed *H. pylori* infection was not the risk of NAFLD development (Table 2). As shown in Table 3, after adjusting for age, sex, SBP, DBP, Scr, BUN, FBG, HbA1C, LDL-C, HDL-C, and triglycerides, *H. pylori*+ was still associated with an increased risk of NAFLD (model 1, *P* < 0.001; model 2, *P* < 0.001; model 3, *P* < 0.001; model 4, *P* = 0.033). Conversely, when other confounding factors (BMI) were further adjusted (model 5), the results showed that there was no appreciable relationship between *H. pylori* infection and NAFLD (*P* = 0.574). Furthermore, Fig. 2 shows that in the subgroups stratified by sex (female: 0.991, *P* = 0.863; male: 1.039, *P* = 0.250), age (< 60: 1.028, *P* = 0.354; ≥ 60: 0.992, *P* = 0.910), BMI (< 25: 1.004, *P* = 0.926; ≥ 25: 1.045, *P* = 0.220), hypertension (No: 1.034, *P* = 0.280; Yes: 0.965, *P* = 0.563), diabetes (No: 1.018, *P* = 0.533; Male: 0.969, *P* = 0.759), and dyslipidemia (No: 1.030, *P* = 0.372; Yes: 1.044, *P* = 0.346), no significant correlation between *H. pylori* positivity and an increased risk of NAFLD was detected.

**Table 2:** The risk of NAFLD development in the univariate and multivariate analyses

Variables	Full sample (n = 71,633)				Matched sample (n = 29,974)			
	Univariate analysis		Multivariate analysis ^a^		Univariate analysis		Multivariate analysis ^b^	
	OR (95%CI)	*P* value	OR (95%CI)	*P* value	OR (95%CI)	*P* value	OR (95%CI)	*P* value
Age	1.02 (1.02–1.02)	< 0.001	1.02 (1.01–1.02)	< 0.001	1.01 (1.01–1.02)	< 0.001	1.01 (1.01–1.02)	< 0.001
Male sex (%)	3.13 (3.03–3.24)	< 0.001	1.09 (1.01–1.18)	0.022	0.35 (0.33–0.36)	< 0.001	0.98 (0.90–1.07)	0.601
BMI (kg/m^2^)	1.62 (1.61–1.63)	< 0.001	1.44 (1.42–1.45)	< 0.001	1.59 (1.57–1.61)	< 0.001	1.42 (1.40–1.44)	< 0.001
Systolic BP (mmHg)	1.03 (1.03–1.03)	< 0.001	1.00 (0.99–1.00)	< 0.001	1.03 (1.03–1.03)	< 0.001	1.00 (0.99–1.00)	0.003
Diastolic BP (mmHg)	1.07 (1.06–1.07)	< 0.001	1.02 (1.02–1.03)	< 0.001	1.06 (1.06–1.06)	< 0.001	1.02 (1.02–1.03)	< 0.001
FBG (mM)	1.46 (1.44–1.49)	< 0.001	1.03 (0.99–1.07)	0.087	1.43 (1.39–1.46)	< 0.001	1.05 (1.01–1.10)	0.018
HbA1C (%)	1.98 (1.92–2.04)	< 0.001	1.23 (1.15–1.30)	< 0.001	1.83 (1.76–1.90)	< 0.001	1.22 (1.13–1.30)	< 0.001
Total cholesterol (mM)	1.45 (1.42–1.47)	< 0.001	–	–	1.35 (1.31–1.39)	< 0.001	–	–^c^
LDL-C (mM)	1.57 (1.53–1.60)	< 0.001	1.35 (1.30–1.40)	< 0.001	1.44 (1.40–1.49)	< 0.001	1.34 (1.28–1.39)	< 0.001
HDL-C (mM)	0.07 (0.07–0.08)	< 0.001	0.36 (0.32–0.39)	< 0.001	0.09 (0.08–0.10)	< 0.001	0.37 (0.33–0.41)	< 0.001
Triglycerides (mM)	2.53 (2.47–2.58)	< 0.001	1.46 (1.42–1.50)	< 0.001	2.32 (2.25–2.39)	< 0.001	1.46 (1.41–1.50)	< 0.001
AST (IU/L)	1.05 (1.04–1.05)	< 0.001	0.97 (0.97–0.97)	< 0.001	1.04 (1.04–1.04)	< 0.001	0.97 (0.97–0.98)	< 0.001
ALT (IU/L)	1.05 (1.05–1.05)	< 0.001	1.03 (1.03–1.03)	< 0.001	1.04 (1.04–1.05)	< 0.001	1.03 (1.03–1.03)	< 0.001
GGT (IU/L)	1.03 (1.03–1.03)	< 0.001	1.00 (1.00–1.00)	< 0.001	1.02 (1.02–1.03)	< 0.001	1.00 (1.00–1.00)	< 0.001
Scr (μM)	1.02 (1.02–1.02)	< 0.001	0.99 (0.99–1.00)	< 0.001	1.02 (1.02–1.02)	< 0.001	1.00 (0.99–1.00)	< 0.001
BUN (mM)	1.11 (1.10–1.13)	< 0.001	0.99 (0.97–1.01)	0.275	1.10 (1.08–1.12)	< 0.001	0.99 (0.97–1.02)	0.546
*H. pylori* ( +)(%)	1.17 (1.14–1.21)	< 0.001	1.02 (0.97–1.08)	0.446	1.01 (0.96–1.06)	0.716	1.00 (0.94–1.06)	0.898

^a^Estimated from Logistic regression analysis and adjusted for Age, sex, BMI, Systolic BP, Diastolic BP, FBG, HbA1C, LDL-C, HDL-C, Triglycerides, AST, ALT, GGT, Scr, BUN and *H. pylori*+ (%)

^b^Estimated from Logistic regression analysis and adjusted for Age, sex, BMI, Systolic BP, Diastolic BP, FBG, HbA1C, LDL-C, HDL-C, Triglycerides, AST, ALT, GGT, Scr, BUN and *H. pylori*+ (%)

^c^Multicollinearity analysis with the significant variables (variance inflation factor test, VIF) was conducted and Total cholesterol was excluded from the multivariate regression model (VIF = 12.104)

*H. pylori*, *Helicobacter pylori*; BMI, body mass index; BP, blood pressure; FBG, fasting blood glucose; HbA1C, glycated hemoglobin; LDL-C, low-density lipoprotein cholesterol; HDL-C, high-density lipoprotein cholesterol; AST, aspartate aminotransferase; ALT, alanine aminotransferase; GGT, gamma-glutamyl transferase; Scr, serum creatinine; BUN, blood urea nitrogen; NAFLD, nonalcoholic fatty liver disease; OR, odd ratio; CI, confidence intervals

**Table 3:** The risk of NAFLD according to the infection of *H. pylori*

	OR^a^	95% CI	*P* value
Model 0	1.196	1.147–1.247	< 0.001
Model 1	1.160	1.110–1.212	< 0.001
Model 2	1.129	1.079–1.181	< 0.001
Model 3	1.112	1.062–1.165	< 0.001
Model 4	1.055	1.004–1.109	0.033
Model 5	1.016	0.962–1.072	0.574

Model 0 is unadjusted

Model 1 is adjusted for age, sex

Model 2 is further adjusted for SBP, DBP, Scr and BUN

Model 3 is further adjusted for FBG and HbA1C

Model 4 is further adjusted for LDL-C, HDL-C, Triglycerides

Model 5 is further adjusted for BMI

^a^Estimated from Logistic regression analysis

*H. pylori*, *Helicobacter pylori*; BMI, body mass index; BP, blood pressure; FBG, fasting blood glucose; HbA1C, glycated hemoglobin; LDL-C, low-density lipoprotein cholesterol; HDL-C, high-density lipoprotein cholesterol; Scr, serum creatinine; BUN, blood urea nitrogen; NAFLD, nonalcoholic fatty liver disease; N, number of subjects; OR, odd ratio; CI, confidence intervals

### Association between different grades of NAFLD and *H. pylori* infection

The relationship between *H. pylori* infection and different grades of NAFLD was further explored. The results showed that *H. pylori* infection is not an independent risk factor for the different grades of NAFLD (mild: OR = 1.014, *P* = 0.619; moderate: OR = 0.976, *P* = 0.668; severe: OR = 0.861, *P* = 0.602) after adjusting for confounding factors (Table 4). In addition, *H. pylori* infection was not a risk factor for liver function damage in patients with non-alcoholic fatty liver disease (AST, *P* = 0.911; ALT, *P* = 0.237; GGT,* P* = 0.776) (Table 5). Regarding lipid metabolism, there were no significant differences in TC (*P* = 0.627), LDL-C (*P* = 0.100), and HDL-C (*P* = 0.746) levels between the *H. pylori*+ and the *H. pylori*− groups among patients with NAFLD. *H. pylori* infection was not a risk factor for liver and kidney damages, abnormal carbohydrate metabolism, or abnormal lipid metabolism in patients with severe NAFLD (*P* > 0.05).

**Table 4:** Odds ratios of *H.pylori* infection in different grades of NAFLD

Variable	*H. pylori−*	*H. pylori*+	Crude OR (95% CI)	*P*	Adjust OR (95% CI)^a^	*P*
NAFLD	14,678 (31.30%)	8617 (34.82%)	1.172 (1.135–1.211)	< 0.001	1.016 (0.962–1.072)	0.574
No	32,210 (68.70%)	16,128 (65.17%)	1.000		1.000	
Mild	11,549 (24.63%)	6711 (27.12%)	1.161 (1.120–1.202)	< 0.001	1.014 (0.960–1.072)	0.619
Moderate	3044 (6.49%)	1852 (7.48%)	1.215 (1.143–1.291)	< 0.001	0.976 (0.875–1.089)	0.668
Severe	85 (0.18%)	54 (0.21%)	1.269 (0.902–1.785)	0.172	0.861 (0.490–1.512)	0.602

^a^Adjusted for Age, sex, BMI, SBP, DBP, Scr, BUN, FBG, HbA1C, HDL-C, LDL-C, Triglycerides

*H. pylori*, *Helicobacter pylori*; BMI, body mass index; BP, blood pressure; FBG, fasting blood glucose; HbA1C, glycated hemoglobin; LDL-C, low-density lipoprotein cholesterol; HDL-C, high-density lipoprotein cholesterol; Scr, serum creatinine; BUN, blood urea nitrogen; NAFLD, nonalcoholic fatty liver disease; OR, odd ratio; CI, confidence intervals

**Table 5:** The relationship between *H.pylori* infection and liver function, renal function, carbohydrate metabolism, lipid metabolism in nonalcoholic fatty liver patients

	NAFLD (23,295)			Mild-NAFLD (18,260)			Moderate-NAFLD (4896)			Severe-NAFLD (139)		
Parameter (n, %)	*H.pylori−* (14,678)	*H.pylori*+ (8617)	*P* value	*H.pylori− *(11,549)	*H.pylori*+ (6711)	*P* value	*H.pylori− *(3044)	*H.pylori*+ (1852)	*P* value	*H.pylori− *(85)	*H.pylori*+ (54)	*P* value
Liver function												
AST increased	893 (6.2%)	529 (6.2%)	0.911	467 (4.1%)	261 (3.9%)	0.582	382 (12.7%)	217 (11.9%)	0.393	20 (24.7%)	11 (21.6%)	0.833
ALT increased	4492 (30.7%)	2572 (29.9%)	0.237	2796 (24.3%)	1456 (21.8%)	< 0.001	1643 (54.2%)	976 (52.9%)	0.391	53 (63.1%)	40 (75.5%)	0.139
GGT increased	3580 (24.8%)	2123 (25.0%)	0.776	2456 (21.6%)	1382 (20.9%)	0.242	1093 (36.5%)	677 (37.0%)	0.712	31 (38.3%)	17 (33.3%)	0.584
Renal function												
Scr increased	53 (0.4%)	25 (0.3%)	0.411	39 (0.3%)	22 (0.3%)	0.995	14 (0.5%)	3 (0.2%)	0.130	0	0	-
BUN increased	824 (5.6%)	550 (6.4%)	0.014	670 (5.8%)	415 (6.2%)	0.299	149 (4.9%)	104 (5.6%)	0.287	5 (6.0%)	4 (7.4%)	0.737
Carbohydrate metabolism												
FBG increased	1097 (8.5%)	810 (10.6%)	< 0.001	727 (7.1%)	532 (9.0%)	< 0.001	361 (13.4%)	262 (15.8%)	0.033	9 (11.1%)	4 (7.7)	0.568
HbA1C increased	1286 (10.7%)	930 (12.8%)	< 0.001	880 (9.3%)	611 (10.8%)	0.003	395 (16.0%)	311 (19.9%)	0.002	11 (18.3%)	8 (22.22%)	0.792
Lipid metabolism												
Total cholesterol increased	1533 (10.5%)	918 (10.7%)	0.627	1149 (10.0%)	679 (10.1%)	0.721	375 (12.3%)	230 (12.4%)	0.929	9 (10.7%)	9 (16.7%)	0.315
LDL-C increased	1114 (7.6%)	706 (8.2%)	0.100	849 (7.4%)	540 (8.1%)	0.087	259 (8.5%)	163 (8.8%)	0.714	6 (7.1%)	3 (5.6%)	1.000
HDL-C decreased	2177 (14.7%)	1291 (15.0%)	0.746	1500 (13.0%)	873 (13.0%)	0.964	652 (21.4%)	398 (21.5%)	0.943	25 (29.8%)	20 (37.0%)	0.457
Triglycerides increased	4953 (33.8%)	3049 (35.4%)	0.012	3433 (29.8%)	2118 (31.6%)	0.010	1484 (48.8%)	902 (48.8%)	0.976	36 (42.9%)	29 (53.7%)	0.226

*H. pylori, Helicobacter pylori;* BMI, body mass index; BP, blood pressure; FBG, fasting blood glucose; HbA1C, glycated hemoglobin; LDL-C, low-density lipoprotein cholesterol; HDL-C, high-density lipoprotein cholesterol; AST, aspartate aminotransferase; ALT, alanine aminotransferase; GGT, gamma-glutamyl transferase; Scr, serum creatinine; BUN, blood urea nitrogen; NAFLD, nonalcoholic fatty liver disease

## Discussion

In this study, we aimed to explore the relationship between *H. pylori* infection and the risk of NAFLD in the Chinese population. Our results showed that 34.5% of the participants were infected with *H. pylori*, and that *H. pylori* positivity was not an independent risk factor for NAFLD after adjusting for age, sex, BMI, and other factors.

The results showed that the levels of BMI, blood pressure, FBG, HbA1c, total cholesterol, LDL-C, and triglycerides were significantly higher in the *H. pylori*+ group than in the negative group, while the level of HDL-C was lower. In recent years, research has been conducted on the association between *H. pylori* infection and unfavorable metabolic characteristics. In a cross-sectional study, 7417 participants were enrolled to assess the relevance between *H. pylori* infection and HbA1c levels. The results indicated that among adult participants without diabetes, *H. pylori* infection demonstrated significant positive correlation with HbA1c level [22]. In addition, a study of 3,578 subjects showed a positive correlation between *H. pylori* infection and metabolic syndrome, especially in women [23]. Although the mechanisms by which *H. pylori* infection relates to metabolic abnormalities have not been fully elucidated, intestinal microflora and their effects on the innate immune system, intestinal endothelial barrier function, production of intestinal metabolites, and fermentation of indigestible carbohydrates could be potential mechanisms.

NAFLD is considered a manifestation of liver metabolic syndrome. In this study, the *H. pylori* positivity rate was higher in the NAFLD group than in the healthy controls (37.9% vs. 33.8%). In recent years, an increasing number of studies have reported a relationship between *H. pylori* infection and NAFLD. A cross-sectional study reported that the infection rate of *H. pylori* in the NAFLD group was significantly higher than that in healthy controls [24]. Another cohort study showed that compared with participants without *H. pylori* infection*, H. pylori* positive patients were at higher risk of developing NAFLD, independent of inflammatory and metabolic risk factors [13]. A recent meta-analysis indicated that in middle-aged individuals, *H. pylori* infection has obvious relevance with increased prevalence and incidence of NAFLD. The review further suggested that more prospective studies and studies investigating mechanisms are essential to better clarify the possible relationship between *H. pylori* infection and NAFLD [25]. Nevertheless, the correlation between them remains controversial. For example, a large retrospective study in Japan claimed that *H. pylori* positivity was not related to NAFLD [14]. Similarly, another retrospective cross-sectional study involving 4,030 Korean participants showed that *H. pylori* infection was not a risk factor for NAFLD based on the hepatic steatosis index [26]. Recently, another cross-sectional study found that there was no significant association between *H. pylori* infection and NAFLD, regardless of age, sex, BMI, and diabetes status [27]. These studies suggest that *H. pylori* may not play a vital role in the development of NAFLD. In our univariate analysis, *H. pylori* infection was an obvious risk factor for the development of NAFLD. Importantly, after adjusting for BMI, lipid profiles, FBG, and HbA1c levels, there was no independent correlation between them. These results suggest that BMI, lipid profiles, FBG, and HbA1c may mediate the association between *H. pylori* infection and NAFLD. *H. pylori* positivity was associated with higher BMI, dyslipidemia, and blood glucose levels, which were significant risk factors for NAFLD.

The existing controversy between *H. pylori* infection and NAFLD may be owing to the different individuals involved in sample populations, and various diagnostic methods used for *H. pylori* infection and NAFLD. For example, studies in Japan, South Korea, and China have proposed different conclusions [13, 26, 28]. These differences may be owing to different dietary patterns, lifestyles, and socioeconomic factors. All of these factors may affect the composition of the commensal gut microbiota. In some studies, the status of *H. pylori* infection was determined by ELISA using serum IgG antibodies against *H. pylori.* However, serological tests do not accurately discriminate between current and past infections [13]. In our current study and other studies, *H. pylori* infection was defined by the urea breath test, which demonstrates current infectivity [18]. Another aspect to consider is the diagnostic method used for NAFLD. In this study, NAFLD was defined by ultrasonography, while other studies defined NAFLD via biopsy or hepatic steatosis index [29]. Besides, a recent study showed that *H. pylori* infection contributes to the progression from NAFLD to non-alcoholic steatohepatitis (NASH) [28]. Taken together, the specific relationship between *H. pylori* infection and NAFLD is worth exploring in the future.

Our study has several limitations. First, this research was a cross-sectional study with its intrinsic restrictions. As such, only an association between *H. pylori* infection status and NAFLD could be suggested and not a cause–effect inference. Second, we used ultrasonography instead of liver biopsies to diagnose NAFLD, which is not sensitive enough to detect mild liver steatosis. However, because of its high sensitivity and specificity for hepatic steatosis examination, this non-invasive method is widely applied in both clinical practice and epidemiological research [30]. Third, a previous study showed that the prevalence of *H. pylori* infection was 66% in rural Chinese populations and 47% in urban Chinese populations, which is higher than that in our study (34.5%) [31]. We did not exclude patients with a history of *H. pylori* eradication or PPI medication owing to the limited clinical data in this study, which may have contributed to the false negatives. However, all included samples were the baseline results of the patient’s first physical examination, which would have reduced the error to some extent. Fourth, although virulence factors of *H. pylori* may influence the degree of NAFLD, they were neglected because individuals undergoing routine health examinations were included in this retrospective study.

In conclusion, our study showed that *H. pylori* infection was not independently associated with NAFLD in China. Further cohort studies involving liver biopsies are required to determine whether *H. pylori* eradication helps to decrease the risk of NAFLD.

## Supplementary Information

**Additional file 1: Figure S1**. Flow diagram of study.

## Data Availability

The raw data generated and analyzed in the current study are not publicly available because of appropriate protection of patient personal information but are available from the corresponding author upon reasonable request.

## Abbreviations

*H. pylori*: *Helicobacter pylori*
BMI: Body mass index
BP: Blood pressure
FBG: Fasting blood glucose
LDL-C: Low-density lipoprotein cholesterol
HDL-C: High-density lipoprotein cholesterol
AST: Aspartate aminotransferase
ALT: Alanine aminotransferase
GGT: Gamma-glutamyl transferase
Scr: Serum creatinine
BUN: Blood urea nitrogen
NAFLD: Nonalcoholic fatty liver disease
^13^C-UBT: ^13^C urea breath test
PSM: Propensity score matching
TC: Total cholesterol
TG: Triglyceride
VIF: Variance inflation factor

## References

[1] Stefan N, Haring HU, Cusi K (2019). Non-alcoholic fatty liver disease: causes, diagnosis, cardiometabolic consequences, and treatment strategies. Lancet Diabetes Endocrinol.

[2] Lee SB, Park GM, Lee JY, Lee BU, Park JH, Kim BG (2018). Association between non-alcoholic fatty liver disease and subclinical coronary atherosclerosis: an observational cohort study. J Hepatol.

[3] Alexander M, Loomis AK, van der Lei J, Duarte-Salles T, Prieto-Alhambra D, Ansell D (2019). Non-alcoholic fatty liver disease and risk of incident acute myocardial infarction and stroke: findings from matched cohort study of 18 million European adults. BMJ.

[4] Lauridsen BK, Stender S, Kristensen TS, Kofoed KF, Kober L, Nordestgaard BG (2018). Liver fat content, non-alcoholic fatty liver disease, and ischaemic heart disease: Mendelian randomization and meta-analysis of 279 013 individuals. Eur Heart J.

[5] Targher G, Byrne CD (2017). Non-alcoholic fatty liver disease: an emerging driving force in chronic kidney disease. Nat Rev Nephrol.

[6] Aron-Wisnewsky J, Vigliotti C, Witjes J, Le P, Holleboom AG, Verheij J (2020). Gut microbiota and human NAFLD: disentangling microbial signatures from metabolic disorders. Nat Rev Gastroenterol Hepatol.

[7] Leung C, Rivera L, Furness JB, Angus PW (2016). The role of the gut microbiota in NAFLD. Nat Rev Gastroenterol Hepatol.

[8] Mouries J, Brescia P, Silvestri A, Spadoni I, Sorribas M, Wiest R (2019). Microbiota-driven gut vascular barrier disruption is a prerequisite for non-alcoholic steatohepatitis development. J Hepatol.

[9] Dunn BE, Cohen H, Blaser MJ (1997). Helicobacter pylori. Clin Microbiol Rev.

[10] Li WQ, Zhang JY, Ma JL, Li ZX, Zhang L, Zhang Y (2019). Effects of Helicobacter pylori treatment and vitamin and garlic supplementation on gastric cancer incidence and mortality: follow-up of a randomized intervention trial. BMJ.

[11] Franceschi F, Zuccala G, Roccarina D, Gasbarrini A (2014). Clinical effects of Helicobacter pylori outside the stomach. Nat Rev Gastroenterol Hepatol.

[12] Cheng DD, He C, Ai HH, Huang Y, Lu NH (2017). The possible role of helicobacter pylori infection in non-alcoholic fatty liver disease. Front Microbiol.

[13] Kim TJ, Sinn DH, Min YW, Son HJ, Kim JJ, Chang Y (2017). A cohort study on Helicobacter pylori infection associated with nonalcoholic fatty liver disease. J Gastroenterol.

[14] Okushin K, Takahashi Y, Yamamichi N, Shimamoto T, Enooku K, Fujinaga H (2015). Helicobacter pylori infection is not associated with fatty liver disease including non-alcoholic fatty liver disease: a large-scale cross-sectional study in Japan. BMC Gastroenterol.

[15] Karami A, Baradaran A (2014). Comparative evaluation of three different methods for HbA1c measurement with high-performance liquid chromatography in diabetic patients. Adv Biomed Res.

[16] Liu WZ, Xie Y, Cheng H, Lu NH, Hu FL, Zhang WD (2013). Fourth Chinese National Consensus Report on the management of Helicobacter pylori infection. J Dig Dis.

[17] Yu YY, Cai JT, Song ZY, Tong YL, Wang JH (2018). The associations among Helicobacter pylori infection, white blood cell count and nonalcoholic fatty liver disease in a large Chinese population. Medicine.

[18] Chen CX, Mao YS, Foster P, Zhu ZW, Du J, Guo CY (2017). Possible association between Helicobacter pylori infection and nonalcoholic fatty liver disease. Appl Physiol Nutr Metab.

[19] Centers for Disease Control and Prevention (2012). Vital signs: awareness and treatment of uncontrolled hypertension among adults–United States, 2003–2010. MMWR Morb Mortal Wkly Rep.

[20] Forbes JM, Cooper ME (2013). Mechanisms of diabetic complications. Physiol Rev.

[21] Kopin L, Lowenstein C (2017). Dyslipidemia. Ann Intern Med.

[22] Chen Y, Blaser MJ (2012). Association between gastric *Helicobacter pylori* colonization and glycated hemoglobin levels. J Infect Dis.

[23] Chen TP, Hung HF, Chen MK, Lai HH, Hsu WF, Huang KC (2015). Helicobacter pylori infection is positively associated with metabolic syndrome in Taiwanese adults: a cross-sectional study. Helicobacter.

[24] Polyzos SA, Kountouras J, Papatheodorou A, Patsiaoura K, Katsiki E, Zafeiriadou E (2013). Helicobacter pylori infection in patients with nonalcoholic fatty liver disease. Metab Clin Exp.

[25] Mantovani A, Turino T, Altomari A, Lonardo A, Zoppini G, Valenti L (2019). Association between *Helicobacter pylori* infection and risk of nonalcoholic fatty liver disease: an updated meta-analysis. Metab Clin Exp.

[26] Baeg MK, Yoon SK, Ko SH, Noh YS, Lee IS, Choi MG (2016). Helicobacter pylori infection is not associated with nonalcoholic fatty liver disease. World J Gastroenterol.

[27] Fan N, Peng L, Xia Z, Zhang L, Wang Y, Peng Y (2018). Helicobacter pylori infection is not associated with non-alcoholic fatty liver disease: a cross-sectional study in China. Front Microbiol.

[28] Sumida Y, Kanemasa K, Imai S, Mori K, Tanaka S, Shimokobe H (2015). Helicobacter pylori infection might have a potential role in hepatocyte ballooning in nonalcoholic fatty liver disease. J Gastroenterol.

[29] Abdel-Razik A, Mousa N, Shabana W, Refaey M, Elhelaly R, Elzehery R (2018). Helicobacter pylori and non-alcoholic fatty liver disease: a new enigma?. Helicobacter.

[30] Lin YC, Chang PF, Chang MH, Ni YH (2018). Genetic determinants of hepatic steatosis and serum cytokeratin-18 fragment levels in Taiwanese children. Liver Int.

[31] Nagy P, Johansson S, Molloy-Bland M (2016). Systematic review of time trends in the prevalence of Helicobacter pylori infection in China and the USA. Gut Pathog.

