# Supplementary figures and images for: Helicobacter pylori infection is not an independent risk factor of non-alcoholic fatty liver disease in China
Source: BMC Gastroenterol. 2022 Feb 24;22:81. doi: 10.1186/s12876-022-02148-6 (PMC8867781; doi:10.1186/s12876-022-02148-6)

## Supplementary Figure 1. Flow diagram of study

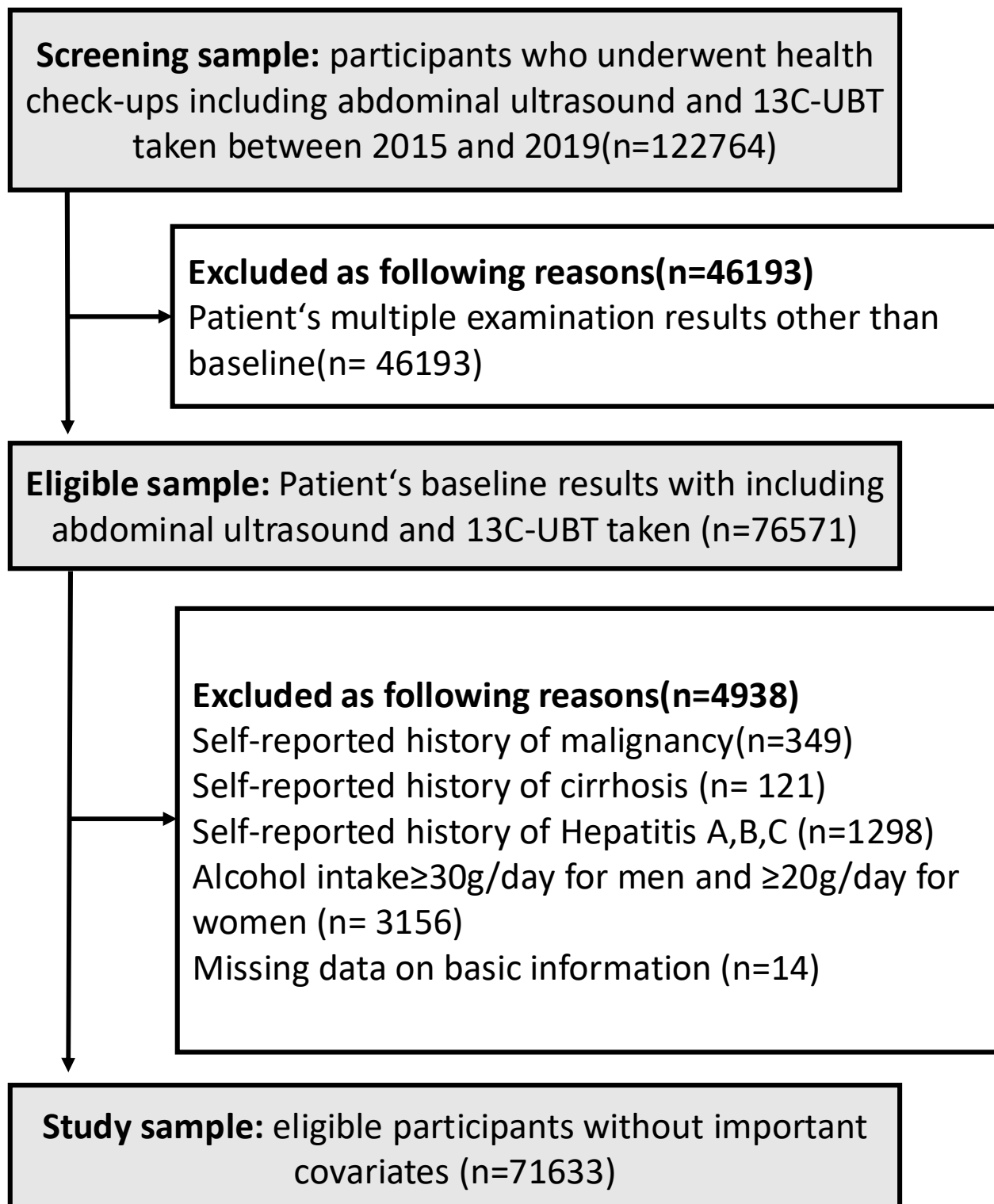

Supplement: Supplementary file 1 — Additional file 1: Figure S1. Flow diagram of study. [file 12876_2022_2148_MOESM1_ESM.pdf]
